# A172 GENETIC AND DIET-ASSOCIATED MUCUS IMPAIRMENTS INCREASE SUSCEPTIBILITY TO ULCERATIVE COLITIS PATHOBIONT-DRIVEN COLITIS.

**DOI:** 10.1093/jcag/gwae059.172

**Published:** 2025-02-10

**Authors:** H Yang, W Kim, c ma, H Yu, B vallance

**Affiliations:** The University of British Columbia Faculty of Medicine, Vancouver, BC, Canada; The University of British Columbia Faculty of Medicine, Vancouver, BC, Canada; The University of British Columbia Faculty of Medicine, Vancouver, BC, Canada; The University of British Columbia Faculty of Medicine, Vancouver, BC, Canada; The University of British Columbia Faculty of Medicine, Vancouver, BC, Canada

## Abstract

**Background:**

The Inflammatory Bowel Diseases (IBD), Crohn’s Disease (CD) and ulcerative colitis (UC) affect > 320,000 Canadians and are increasing in incidence. Ileal CD has been linked to the overgrowth of mucosal adherent-invasive *Escherichia coli*. Recent studies have also implicated the adherence of *E. coli* pathobionts to the colonic mucosa of UC patients. Using the representative UC *E. coli* pathobiont p19A, we recently demonstrated it aggravated chemical-induced colitis in susceptible mice, through the actions of the toxin alpha-hemolysin, and by adhering to the inflamed colonic mucosa via the adhesin FimH. It is less clear what host factors control susceptibility to UC pathobionts. I **hypothesize** that a key susceptibility factor is the glycosylated mucin Muc2, which forms the protective mucus layer that covers the colonic epithelium and is often impaired in UC patients.

**Aims:**

To define the role of colonic mucus structure and function in determining susceptibility to the p19A pathobiont, and its ability to cause colitis in mice.

**Methods:**

Susceptibility to p19A was tested in two mouse models of colonic mucus impairment. The first is a mouse strain deficient in core 1 derived O-glycans in their intestinal epithelial cells (IEC *C1galt1*^-/-^), resulting in reduced mucin glycosylation, and thus a thin and impaired mucus barrier. The second model involves feeding wildtype (WT) C57BL/6J mice a fiber-free (FF) diet, resulting in a significantly thinner colonic mucus layer. The mice were subsequently orally gavaged with p19A and their susceptibility determined by assaying p19A burdens, intestinal histopathology, inflammatory cytokine production.

**Results:**

Following oral gavage of WT mice fed a normal diet, immunostaining identified p19A localizing within the colonic mucus layer, but it did not reach the epithelium or cause disease. In contrast, p19A regularly reached the colonic epithelial surface of mucus-impaired IEC *C1galt1*^-/-^ mice (as compared to IEC *C1galt1*^flox/flox^ mice) and in WT mice fed a fiber-free diet. This epithelial adherence was associated with increased p19A burdens, histopathology and production of inflammatory markers such as TNF-_a_ and lipocalin 2. Notably, fiber-free diet-fed mice showed reduced SCFA levels in their feces and thinner mucus layer in their colon at baseline.

**Conclusions:**

Our results indicate that UC *E. coli* pathobionts are able to reach the colonic epithelial surface and induce colitis in hosts suffering genetic or diet-based mucus dysfunction. This susceptibility highlights the important role played by intestinal mucus in protecting against IBD.

**Funding Agencies:**

CCC, CIHR

